# Genetic and epigenetic landscape of O^6^-methylguanine-DNA methyltransferase (MGMT): implications for DNA repair and cancer therapeutics

**DOI:** 10.37349/etat.2025.1002335

**Published:** 2025-08-28

**Authors:** Shishir Singh, Rajeev Nema, Monisha Banerjee, Atar Singh Kushwah

**Affiliations:** European University Cyprus, Cyprus; ^1^Department of Gastroenterology, Icahn School of Medicine at Mount Sinai, New York, NY 10029, USA; ^2^Department of Biosciences, Manipal University, Jaipur 303007, India; ^3^Molecular and Human Genetics Laboratory, Department of Zoology, University of Lucknow, Lucknow 226007, India; ^4^Women’s Biomedical Research Institute, Icahn School of Medicine at Mount Sinai, New York, NY 10029, USA

**Keywords:** O^6^-Methylguanine-DNA methyltransferase (MGMT), DNA repair mechanisms, genetic and epigenetic modifications, precision oncology, CRISPR/Cas9, PARP inhibitors

## Abstract

O^6^-Methylguanine-DNA methyltransferase (MGMT) acts as a genomic custodian, reversing alkylation damage to preserve DNA integrity. However, when its regulatory balance tips via promoter methylation, polymorphisms, or epigenetic silencing, MGMT can become a liability, fuelling cancer progression, treatment resistance, and poor outcomes across malignancies. This review uncovers the nuanced control of MGMT, revealing how its genetic and epigenetic shifts shape tumor behavior, therapeutic response, and risk stratification. We aim to transform molecular insights into actionable clinical strategies, reimagining MGMT as both a biomarker and therapeutic lever. We curated high-impact studies (up to 2025) from PubMed, Scopus, and Web of Science, focusing on MGMT modulation, synthetic lethality, CRISPR-based restoration, and epigenetic therapies. Emerging multi-omics and translational frameworks were prioritized. MGMT’s activity is choreographed by an intricate interplay of promoter methylation, histone marks, transcriptional regulation, and microRNA influence. These dynamics critically affect sensitivity to alkylating agents like temozolomide. Intriguingly, MGMT also engages with the immune landscape modulating response to immunotherapies. Innovations in multi-omics, single-cell analytics, and AI-based biomarker profiling are unveiling previously hidden regulatory layers. Decoding MGMT’s regulation unlocks new therapeutic frontiers. Cutting-edge strategies from CRISPR to liquid biopsy promise more personalized, resistance-proof cancer care.

## Graphical abstract

**Graphical abstract.**
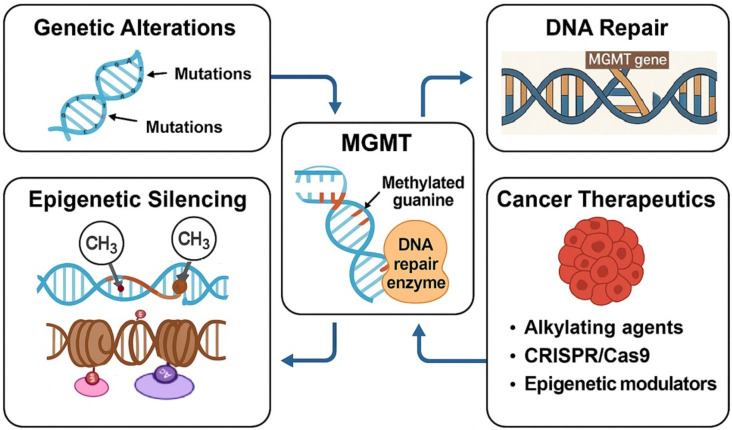
** MGMT in cancer: from genetic and epigenetic regulation to therapeutic targeting**. MGMT: O^6^-methylguanine-DNA methyltransferase

## Introduction

O^6^-Methylguanine-DNA methyltransferase (MGMT) is a critical DNA repair enzyme responsible for maintaining genomic integrity by removing alkyl adducts from the O^6^ position of guanine, a common site of damage from both endogenous and therapeutic alkylating agents. MGMT functions through a unique single-step damage reversal process, transferring the alkyl group to a cysteine residue in its active site, thereby restoring the DNA and simultaneously inactivating itself earning its classification as a “suicide” enzyme [1, 2].

This repair mechanism is essential for preventing mutagenic lesions such as G:C to A:T transitions that arise when O^6^-methylguanine (O^6^-MeG) mispairs with thymine during replication. Such mutations, if unrepaired, can lead to chromosomal instability, cellular transformation, and apoptosis [3]. Thus, MGMT serves a protective role in normal tissues. However, this same repair activity becomes a double-edged sword in cancer therapy. Tumors with high MGMT expression often exhibit resistance to alkylating chemotherapeutic agents such as temozolomide (TMZ), carmustine (BCNU), and lomustine (CCNU), which rely on unrepaired DNA lesions to trigger cancer cell death [4, 5].

The expression and function of MGMT are regulated both genetically and epigenetically. Promoter hypermethylation, an epigenetic mechanism that silences gene transcription, is frequently observed in gliomas and other solid tumors. This silencing correlates with decreased MGMT protein levels and increased tumor sensitivity to alkylating agents, making *MGMT* promoter methylation a useful prognostic and predictive biomarker [5, 6]. On the other hand, certain polymorphisms, such as rs12917 and rs2308327, have been linked to altered MGMT expression or repair efficiency and may influence individual susceptibility to cancer or treatment response [6].

In light of these findings, several therapeutic strategies have been developed to modulate MGMT activity. MGMT inhibitors like O^6^-benzylguanine (O^6^-BG) have been tested in clinical and preclinical settings to overcome drug resistance, though toxicity to healthy tissues remains a concern [2, 4]. Ultimately, understanding MGMT’s dual role offers critical insights for personalized cancer therapy, especially in malignancies where alkylating agents remain a standard of care. Despite extensive research into MGMT’s role in DNA repair and chemotherapy resistance, a critical integration of how its genetic polymorphisms and epigenetic modifications collectively influence treatment outcomes across cancer types is lacking. Additionally, recent advances in genome editing, epigenetic therapeutics, and molecular profiling call for an updated synthesis that connects molecular mechanisms to clinical translation.

In this review, we take a comprehensive view of the intricate regulatory landscape surrounding MGMT in cancer, aiming to unravel its multifaceted biological and clinical relevance. We delve into the genetic and epigenetic frameworks that shape MGMT expression, illuminating their consequences for genomic stability, tumor dynamics, and resistance to therapy. We further investigate MGMT’s emerging role as a molecular interlocutor within the tumor immune microenvironment (TIME), revealing its influence on the broader cellular ecosystem. Finally, we explore the translational potential of MGMT as a biomarker, highlighting its promise in guiding clinical decisions and in the evolution of liquid biopsy technologies. By synthesizing insights from across malignancies, our goal is to deepen our understanding of MGMT’s prognostic and therapeutic impact and to chart a path toward its more effective integration into precision oncology.

## *MGMT* gene: structure, function, and regulation

### Gene structure and function

The *MGMT* gene is crucial for DNA repair. It protects cells by reversing DNA damage caused by alkylating agents chemicals that add harmful methyl or ethyl groups to DNA. MGMT specifically removes the alkyl group from the O^6^ position of guanine, a site that, if left unrepaired, can cause incorrect base pairing, mutations, and eventually cancer [7]. This reaction occurs in a single step where MGMT transfers the alkyl group to its own cysteine residue, resulting in its inactivation. For this reason, MGMT is often referred to as a “suicide” repair enzyme [2].

The *MGMT* gene is located on chromosome 10q26 and consists of 5 exons and 4 introns and spans greater than 300 kb [7]. MGMT protein has 207 amino acids and has many conserved regions throughout. Structurally, MGMT has a conserved DNA-binding domain and an active site that includes a cysteine at position 145, essential for its repair function [7]. Although MGMT is expressed in most normal tissues, the level of expression can vary significantly, influencing how cells respond to DNA damage.

**Figure 1:**
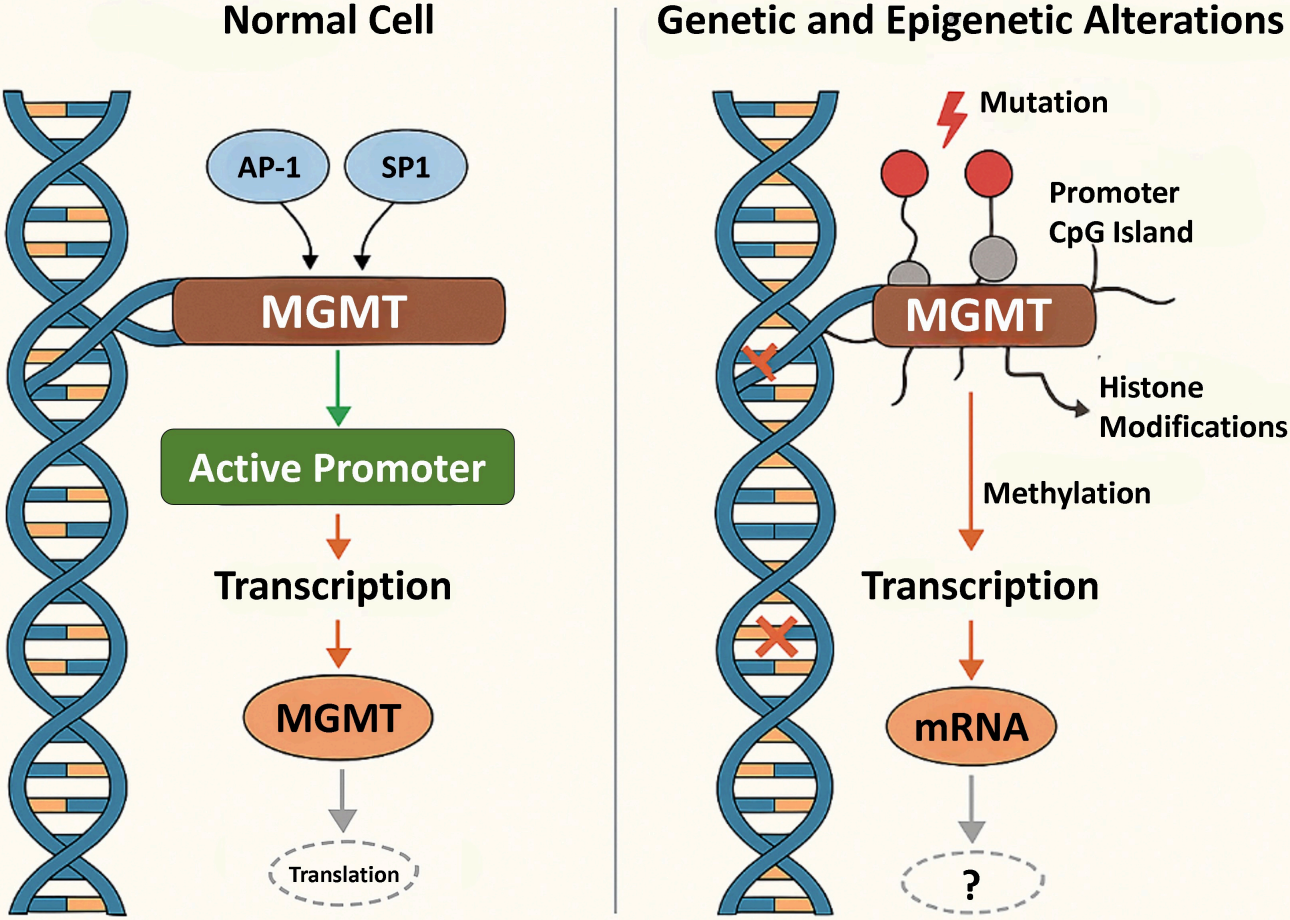
**Mechanistic overview of *MGMT* gene regulation in normal and cancerous cells.** AP-1: activator protein-1; *MGMT*: O^6^-methylguanine-DNA methyltransferase

It has repetitive GC-rich sequences comprising a CpG island. The promoter region of *MGMT* spans 1.2 kb and includes the first exon and part of the first intron [7]. Expression of *MGMT* can be induced mainly by DNA damage, glucocorticoids, cyclic AMP, protein kinase C and interaction of several transcriptional factors like SP1, activator proteins 1 and 2 (AP-1 and AP-2) with its promoter region. MGMT functions as a transferase and an alkyl-group acceptor (Figure 1). MGMT recognizes alkyl DNA adducts at the O^6^ position of guanine and transfers the alkyl moiety to a cysteine residue within its own structure, effectively repairing lesions in a stoichiometric and irreversible manner [8]. While its primary substrate is O^6^-MeG, MGMT is also capable of repairing larger alkyl adducts such as O^6^-ethylguanine and O^4^-methylthymine, indicating its versatility beyond standard substrates [9]. Interestingly, the base excision repair (BER) mechanism addresses other types of alkyl damage, specifically N7-methylguanine and N3-methyladenine, whereas MGMT focuses predominantly on O^6^ and O^4^ adducts [10].

The repair of O^6^-MeG lesions can lead to G:T mismatches, which can subsequently be rectified through mismatch repair (MMR) mechanisms [11]. It is well documented that downregulation of the *MGMT* gene is reported in a variety of tumor types, including gliomas, colorectal cancers, and lung cancers. This loss significantly contributes to tumorigenesis by facilitating the persistence of mutations resulting from mispairing during replication [12, 13]. Therefore, MGMT plays a crucial role in protecting cells from mutagenic DNA adducts, and its diminished expression is associatively linked to various malignancies. This underscores its importance as a biomarker in cancer prognosis and therapy [13, 14].

### Regulatory mechanisms of MGMT expression

The expression of MGMT is tightly regulated at multiple levels, which influences both cancer development and treatment response. Abnormal MGMT regulation is often observed in cancers like glioblastoma, colorectal, lung, and cervical cancer, where it affects sensitivity to alkylating chemotherapies [15, 16].

MGMT expression is orchestrated by promoter activity, anchored in a CpG island, an epigenetic hotspot dense with cytosine-guanine sequences that acts as a regulatory epicenter (Figure 1). In many cancers, this region becomes hypermethylated, leading to transcriptional silencing of MGMT and making tumor cells more responsive to alkylating agents like TMZ [4, 17]. Histone modifications, particularly trimethylation of histone H3 at lysine 9 (H3K9me3), also suppress MGMT expression by condensing the chromatin structure and making it inaccessible for transcription [18].

Transcription factors such as AP-1 and SP1 usually enhance *MGMT* expression by binding to its promoter. In contrast, p53 may either repress or activate MGMT depending on the context, highlighting its complex regulatory role [19, 20]. Non-coding RNAs (ncRNAs), especially microRNAs (miRNAs), regulate MGMT after transcription by binding to its mRNA and blocking translation or promoting degradation, further fine-tuning its protein levels in cells [5].

## Genetic variants of *MGMT* and their role in cancer

### *MGMT* gene variants and cancer susceptibility

Gene variants in the *MGMT* gene can significantly affect its DNA repair efficiency, contributing to individual cancer risk. These single-nucleotide polymorphisms (SNPs) may influence protein expression, structure, or function and are often investigated as biomarkers of cancer susceptibility.

One well-studied variant is rs12917 (C>T)**,** located in exon 5 of the *MGMT* gene. This polymorphism has been associated with reduced MGMT activity, which limits the cell’s ability to repair O^6^-MeG lesions effectively. The resulting accumulation of DNA damage increases the risk of tumor initiation, particularly in tissues frequently exposed to genotoxic agents, such as the colon and lungs [6].

Another important SNP is rs2308321 (T>C), a nonsynonymous change that leads to an amino acid substitution. This variant has been linked to increased tumor progression in several cancers. It may alter the three-dimensional structure of the MGMT protein, affecting its stability or ability to interact with damaged DNA substrates [2]. Evidence suggests that individuals carrying the C allele may exhibit poorer outcomes when treated with alkylating agents due to altered MGMT activity.

Additionally, the rs16906222 (A>G) variant, found in the promoter region, is believed to impact chromatin accessibility. This alteration can reduce transcription factor binding efficiency and thus lower *MGMT* gene expression. A decrease in expression can sensitize tumors to alkylating drugs but may also lead to increased baseline mutation rates [5].

### MGMT mutations in tumorigenesis

Beyond inherited polymorphisms, somatic mutations in the *MGMT* gene are increasingly observed in several malignancies including glioblastoma and colorectal carcinoma. These mutations are often acquired during tumor evolution and lead to loss-of-function changes in the MGMT protein [2]. Without functional MGMT, cells lose a critical mechanism for repairing alkylating DNA damage, resulting in genomic instability.

Such instability promotes further mutations in tumor suppressors and oncogenes, accelerating tumor progression. Interestingly, tumors harboring inactivating MGMT mutations often become more sensitive to chemotherapy, particularly alkylating agents, since they lack an effective repair mechanism [3, 19]. However, this also increases the risk of developing secondary malignancies or drug resistance through alternative repair pathways.

## Epigenetic modifications of MGMT in cancer

Epigenetic mechanisms play a central role in regulating the expression of MGMT, a key DNA repair enzyme. Unlike genetic mutations, epigenetic changes do not alter the DNA sequence but instead modify how genes are expressed. These changes can significantly influence cancer progression and treatment response by silencing or activating genes like *MGMT*. The three main epigenetic mechanisms impacting MGMT are promoter methylation, histone modifications, and regulation by ncRNAs.

### *MGMT* promoter methylation in tumor progression

Promoter hypermethylation is one of the most studied epigenetic silencing mechanisms of MGMT. This process involves the addition of methyl groups to CpG islands in the gene’s promoter region, leading to chromatin condensation and transcriptional repression. *MGMT* promoter methylation has been observed across several cancers and is often linked to enhanced treatment response due to impaired DNA repair capacity [16, 17].

In glioblastoma multiforme (GBM), *MGMT* promoter methylation is a well-established biomarker for predicting the effectiveness of the alkylating agent TMZ. Patients whose tumors have methylated *MGMT* promoters often show better responses to TMZ and improved overall survival, since the absence of MGMT prevents repair of TMZ-induced DNA lesions [2, 16].

In colorectal cancer, *MGMT* promoter methylation is frequently associated with microsatellite instability (MSI), a condition where DNA MMR is impaired. *MGMT* silencing leads to the accumulation of mutations, contributing to tumorigenesis and resistance to DNA-damaging treatments [15].

In cervical cancer, *MGMT* promoter methylation has been correlated with radiation sensitivity. The loss of MGMT-mediated DNA repair may make tumor cells more vulnerable to DNA strand breaks induced by radiotherapy, offering a possible explanation for better treatment outcomes in methylation-positive patients [6].

### Histone modifications and *MGMT* silencing

Apart from DNA methylation, MGMT expression is also regulated by post-translational modifications of histone proteins. These modifications alter chromatin structure, influencing gene accessibility.

Two key repressive marks, H3K9me3 and histone H3 lysine 27 trimethylation (H3K27me3) are particularly involved in *MGMT* silencing. High levels of these marks around the *MGMT* promoter region led to tightly packed chromatin, which blocks RNA polymerase from accessing the gene, thereby halting transcription [2, 18].

These histone modifications often work in conjunction with DNA methylation, creating a robust silencing environment that further diminishes MGMT expression. The loss of MGMT reduces the cell’s capacity to repair O^6^-alkylguanine lesions, increasing susceptibility to mutagenesis and, paradoxically, to chemotherapy agents that rely on such damage for their cytotoxic effects.

### ncRNAs and MGMT regulation

Emerging evidence highlights the role of ncRNAs, particularly miRNAs in the post-transcriptional regulation of MGMT. These small RNAs bind to the 3′ untranslated region (3′UTR) of *MGMT* mRNA, blocking its translation or leading to degradation.

Two miRNAs, miR-181c and miR-648, have been shown to directly target *MGMT* mRNA, decreasing protein production. This downregulation exacerbates DNA repair deficiencies in cancer cells and enhances sensitivity to DNA-damaging agents like TMZ and radiotherapy [2, 5]. High expression levels of these miRNAs are often observed in tumors with low MGMT protein, suggesting a potential feedback loop between ncRNA activity and chemotherapeutic response.

In addition to miR-181c and miR-648, other miRNAs have also been implicated in MGMT regulation. For instance, miR-370-3p has been shown to suppress MGMT expression in glioma by targeting its mRNA, contributing to TMZ resistance and malignant progression [21]. Similarly, miR-221-3p downregulates MGMT in hepatocellular carcinoma, thereby promoting tumorigenesis [22]. These findings highlight the tissue-specific regulatory roles of ncRNAs and reinforce their potential as both therapeutic targets and biomarkers across different cancer types.

Targeting these miRNAs or their interaction with *MGMT* mRNA may open new therapeutic avenues for overcoming drug resistance, particularly in glioblastoma and colorectal cancer, where MGMT activity critically determines treatment outcome.

## MGMT expression and TIME

**Figure 2:**
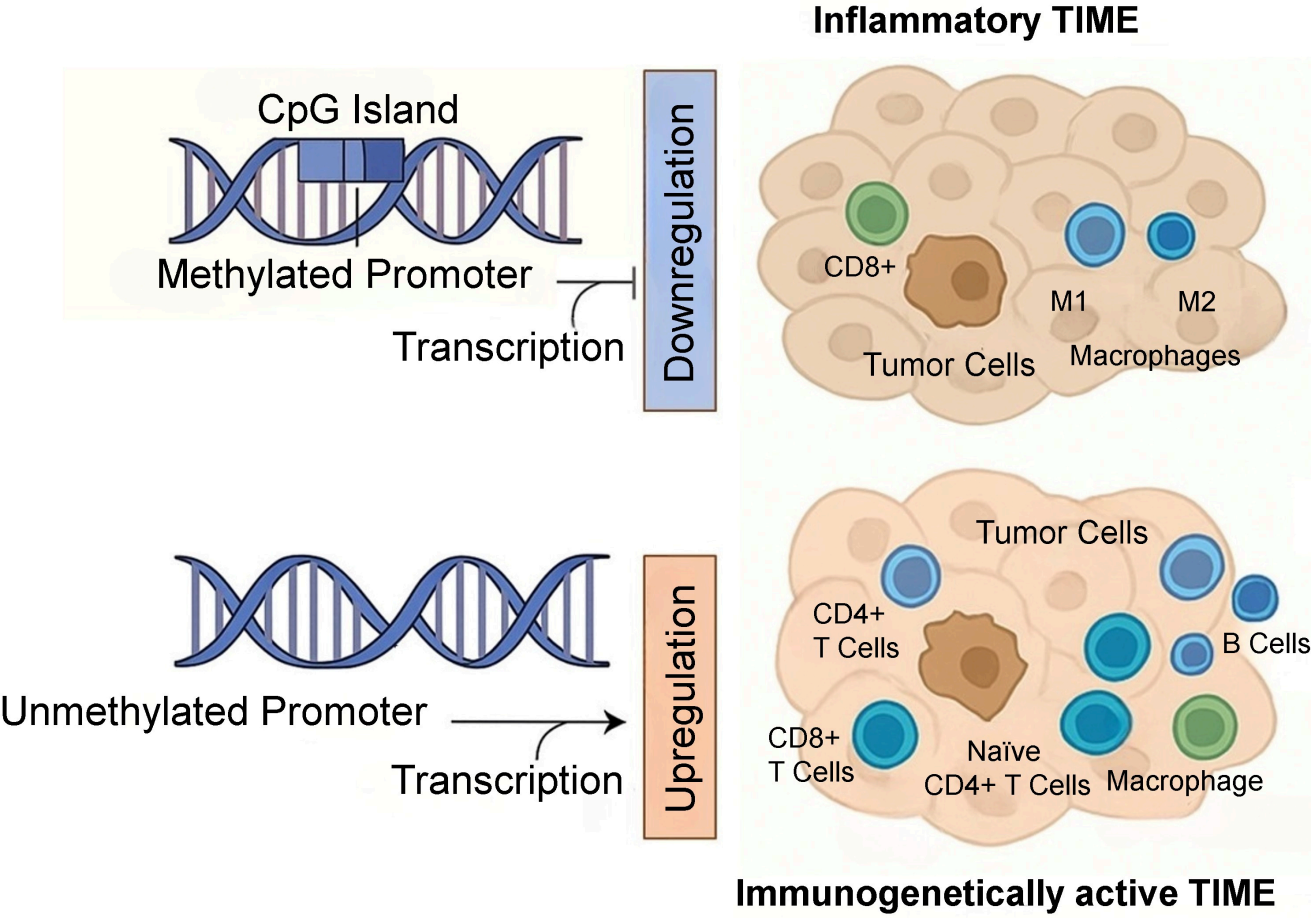
Epigenetic regulation of MGMT expression and its impact on tumor immune microenvironment (TIME)

Recent studies suggest that MGMT expression not only affects tumor sensitivity to therapy but also plays a nuanced role in shaping the TIME, particularly in glioblastoma and other aggressive malignancies [23–25] (Figure 2). Interestingly, high MGMT expression has been linked to an immunologically active TIME. For instance, Kushihara et al. [23] showed that glioblastomas with elevated MGMT levels were enriched with tertiary lymphoid structures (TLS) specialized immune cell aggregates associated with enhanced anti-tumor immunity and improved response to immune checkpoint inhibitors. This supports the view that MGMT may contribute to immune surveillance mechanisms, potentially correlating with better outcomes in certain patient subgroups [26, 27].

On the flip side, low MGMT expression is often seen in tumors that respond favorably to alkylating chemotherapy. This heightened chemosensitivity leads to increased tumor cell apoptosis, resulting in the release of tumor antigens and the recruitment of immune cells such as cytotoxic T lymphocytes (CTLs) [24, 28]. Such a pro-inflammatory milieu may amplify anti-tumor immunity and improve prognosis [29, 30]. This differential MGMT expression contributes to shaping either an inflammatory or immunogenetically active TIME, as shown in Figure 2.

Moreover, MGMT expression appears to modulate immune cell infiltration. Low MGMT levels are frequently associated with higher infiltration of CD8^+^ T cells, which correlates with a favorable prognosis [25]. In contrast, high MGMT expression may attract regulatory T cells (Tregs), foster an immunosuppressive microenvironment and aid immune evasion [31, 32].

Notably, intratumoral heterogeneity in MGMT expression further complicates this relationship. Distinct tumor regions may exhibit variable MGMT levels, leading to localized differences in immune cell activity, chemoresistance, and immunotherapeutic response [33, 34]. MGMT-positive subpopulations, in particular, may confer treatment resistance while dampening local immune activation [35, 36].

Together, these insights highlight the dualistic nature of MGMT not only as a DNA repair factor but also as a modulator of immune dynamics within the tumor niche. A deeper understanding of this interplay could inform personalized treatment strategies, integrating MGMT status with immunotherapeutic approaches to improve outcomes in glioblastoma and other refractory cancers.

In cervical cancer, *MGMT* silencing is a common feature, occurring within an immune environment shaped by genetic variations in cytokine genes. These variants influence pro- and anti-inflammatory signaling, helping to define the tumor’s biological context. Our team has previously identified specific polymorphisms in TNF-α, IL-10, and IL-6 as contributors to increased cervical cancer risk and diminished response to cisplatin-based chemoradiotherapy (CRT) [37, 38]. By integrating MGMT expression profiling with these immune-related genetic markers, we may be able to more accurately stratify patients and predict treatment outcomes. Additionally, machine learning models combining cytokine SNP data with clinical variables have shown promise in forecasting cancer susceptibility and immune responsiveness [39], pointing toward a powerful intersection between computational analytics and biomarker-guided immuno-oncology.

## MGMT and chemo/radiotherapy and its resistance

The ability of tumor cells to repair chemotherapy-induced DNA damage plays a central role in treatment resistance. MGMT, a DNA repair protein, is one of the most studied factors in this context. MGMT can directly reverse the DNA damage caused by alkylating agents, reducing the effectiveness of chemotherapy. Its expression level and epigenetic status have significant implications for cancer therapy outcomes, especially in glioblastoma and other solid tumors.

The *MGMT* gene plays a significant role in the context of chemotherapy and radiotherapy, particularly in the treatment of GBM and other malignancies requiring alkylating agents like TMZ. MGMT is primarily responsible for repairing O^6^-MeG, a toxic DNA adduct that can arise from exposure to chemotherapy. The efficacy of alkylating chemotherapeutics is often compromised in tumors exhibiting high levels of MGMT expression due to its DNA repair capabilities [40, 41]. Consequently, the status of MGMT is a critical determinant of treatment resistance or sensitivity and overall patient prognosis.

The molecular mechanisms underlying acquired resistance to TMZ are complex, involving both pre-existing and treatment-acquired modifications in MGMT expression and activity. Research demonstrates that low pre-treatment levels of MGMT protein are associated with a better response to alkylating agents; in contrast, elevated MGMT activity correlates with primary resistance [40, 42]. Specifically, studies have indicated that tumors with significant MGMT expression levels exhibit diminished responsiveness to TMZ [40]. Conversely, in cases of recurrent GBM, rapid alterations in the methylation status of MGMT, wherein previously methylated promoters become unmethylated, have been implicated in the development of resistance [42].

In addition to MGMT levels, other factors contribute to the intricate drug resistance landscape. For instance, the cellular microenvironment, including hypoxia, can affect MGMT activity and contribute to chemoresistance in GBM. Hypoxia-inducible factors have been shown to mediate the expression of MGMT, thereby enhancing resistance to TMZ [43, 44]. Furthermore, autophagy is an emerging factor that may also play a role in TMZ resistance, as it has been associated with the modulation of DNA repair processes involving MGMT [45].

*MGMT* promoter methylation status remains one of the most critical biomarkers for predicting response to TMZ treatment. Clinical studies highlight that the presence of methylation can significantly improve outcomes, making it an important predictive factor in treatment planning for GBM patients [46, 47]. The relationship between *MGMT* promoter methylation and treatment response indicates that these alterations can serve as both prognostic and predictive markers [42, 48].

Advancements in therapy have also focused on strategies to combat MGMT-mediated resistance. Studies have explored combining TMZ with agents that inhibit MGMT activity or downregulate its expression. For instance, approaches utilizing cold atmospheric plasma to restore sensitivity to chemotherapy in MGMT-expressing tumor cells have shown promise, indicating a potential avenue for enhancing treatment efficacy [49]. Additionally, adjunctive therapies, including inhibitors of histone deacetylation, have been implicated in suppressing multidrug resistance (MDR) mechanisms by downregulating MGMT expression [50] and thus enhancing the cytotoxicity of alkylating agents [41].

Research continues to explore novel avenues to augment sensitivity to alkylating agents in MGMT-expressing tumors. For instance, targeting pathways that regulate the stability of mRNA transcripts associated with MGMT may prove beneficial in decreasing its levels and facilitating chemosensitivity [45]. There are also considerations regarding personalized medicine approaches, where MGMT expression status is evaluated prior to the initiation of therapy to tailor treatments effectively [51, 52].

Overall, MGMT’s role in resistance to chemotherapy and radiotherapy underscores the necessity for tailored therapeutic strategies in oncology, particularly for glioblastoma patients. Continued exploration of the molecular underpinnings involved provides insights into overcoming drug resistance and improving prognosis with the use of epigenetic modifications and novel therapeutic combinations.

### Role in alkylating agent resistance

Resistance to alkylating agents such as TMZ and BCNU is closely linked to the DNA repair activity of MGMT. Tumors with high MGMT expression can effectively reverse therapy-induced DNA damage, limiting drug efficacy and promoting cell survival [16, 53, 54]. Elevated MGMT activity in tumors has been recognized as a significant mechanism behind resistance to alkylating therapies, allowing malignant cells to efficiently repair alkylation damage before it induces lethal replication errors [55].

The prognostic significance of MGMT in GBM is well-documented, particularly regarding its promoter methylation status. Methylation of the *MGMT* promoter leads to gene silencing and decreased expression of the MGMT protein, which correlates with diminished DNA repair capability and increased sensitivity to TMZ treatment. Patients with GBM exhibiting a methylated *MGMT* promoter tend to have better treatment outcomes, as they are less able to proficiently repair the damage caused by alkylating agents, compared with those possessing unmethylated promoters who often show poor responses [16, 55, 56].

Gupta et al. [6] demonstrated that tumor-specific *MGMT* promoter methylation serves both as a prognostic biomarker and as a predictive factor for therapeutic response. For instance, the EORTC-NCIC trial highlighted the correlation between *MGMT* promoter methylation and favourable treatment outcomes following TMZ and radiotherapy, reinforcing the utility of this biomarker in guiding clinical treatment decisions [55, 57]. Additionally, studies indicate that higher levels of methylation within the *MGMT* promoter region are associated with improved overall survival rates in GBM patients, underscoring the need for routine assessment of MGMT status in neuro-oncology [16, 58, 59].

In summary, alkylating agents such as TMZ and BCNU induce DNA damage through O^6^-alkylation of guanine. The MGMT enzyme mitigates this damage via DNA repair; thus, high expression levels of MGMT can lead to treatment resistance. Conversely, *MGMT* promoter methylation silences the expression of this enzyme, enhancing sensitivity to TMZ and correlating with improved patient outcomes. This dynamic positions MGMT methylation as both a prognostic and predictive biomarker essential for patient stratification in glioblastoma therapies [60–63] (Table 1).

**Table 1:** MGMT-targeted strategies in cancer

**Strategy**	**Mode of action**	**Remarks/Examples**
Direct MGMT inhibitors	Irreversibly binds and inactivates MGMT to deplete its activity	O^6^-Benzylguanine (O^6^-BG); enhances TMZ efficacy but may cause myelotoxicity
Epigenetic silencing	Induce *MGMT* promoter methylation or alter chromatin to reduce transcription	Use of DNMT inhibitors (decitabine) or HDAC inhibitors (vorinostat)
RNA-based approaches	Use siRNA, shRNA, or ASOs to degrade *MGMT* mRNA or block translation	Experimental use of MGMT siRNA or antisense oligonucleotides like ALZ003
CRISPR/Cas9-mediated knockout	Gene editing to remove *the MGMT* gene function	Preclinical models to study TMZ sensitization
Targeted drug delivery	Deliver MGMT inhibitors selectively to tumor cells to minimize toxicity	Nanoparticles or liposomes loaded with O^6^-BG or MGMT siRNA

DNMT: DNA methyltransferase; MGMT: O^6^-methylguanine-DNA methyltransferase; TMZ: temozolomide; HADC: histone deacetylase

### Synthetic lethality approaches

Recent reports highlight the synthetic lethality, a strategy that targets vulnerabilities in tumor cells that lack functional MGMT. It plays a crucial role in the repair of DNA damage caused by alkylating agents such as TMZ and BCNU, which modify the O^6^ position of guanine and can lead to replication errors and cell death if unrepaired [64, 65]. In tumors where MGMT is overexpressed, these repair mechanisms can confer resistance to overcome this; one promising strategy involves the combination of MGMT inhibitors (e.g., O^6^-BG) with poly(ADP-ribose) polymerase (PARP) inhibitors (e.g., olaparib, niraparib), which disrupt parallel DNA repair pathways. This dual inhibition causes the accumulation of DNA damage and enhances tumor cell death, particularly in MGMT-deficient settings [66–68]. For instance, studies reveal that inhibiting PARP activity in conjunction with the removal of MGMT allows for heightened sensitivity to TMZ by diminishing the tumor’s ability to repair alkylated DNA. Specifically, PARP inhibitors can reduce the ability of MGMT to repair O^6^-MeG lesions, effectively enhancing the cytotoxic effects of alkylating agents [69]. Additionally, the use of ATR (ataxia telangiectasia and Rad3-related protein) inhibitors alongside PARP inhibitors has been shown to further intensify replication stress and drive tumor cell apoptosis in certain cancer models [66, 70].

Furthermore, the strategic combination of PARP and MGMT inhibitors is being explored in various cancers that exhibit similar resistance mechanisms [71, 72]. Preclinical data suggest that the disruption of both DNA repair pathways exploits the vulnerabilities of certain tumors and holds potential for improving patient outcomes in challenging clinical contexts, including those with mutant or hyperactive MGMT profiles [64, 68].

In summary, the strategy of utilizing synthetic lethality through the combination of MGMT inhibitors and PARP inhibitors represents a promising avenue for circumventing drug resistance in MGMT-overexpressing tumors, facilitating more effective treatment regimens and potentially leading to better clinical outcomes in patients afflicted with these malignancies.

### MGMT status as a prognostic marker

The methylation status of the *MGMT* promoter has emerged as a critical biomarker in the management of GBM and other malignancies. Numerous studies underscore its dual role as both a predictive marker for treatment response and a prognostic marker for overall survival. This understanding is integral to the concept of precision medicine, where therapeutic strategies are tailored based on the molecular characteristics of a patient’s tumor.

Research has consistently demonstrated that *MGMT* promoter methylation is associated with enhanced sensitivity to alkylating agents, particularly TMZ in GBM. In the article by Hegi et al. [16], they conducted seminal work showing that patients with methylated *MGMT* promoters exhibited significantly better survival outcomes when treated with TMZ compared to those with unmethylated MGMT, who often showed rapid tumor progression and limited response to therapy. This study has laid the groundwork for incorporating MGMT methylation testing into clinical decision-making [16].

Esteller et al. [15] also established the significance of *MGMT* promoter methylation as a marker of cancer-specific prognosis, demonstrating that gene silencing through methylation leads to reduced MGMT expression and an augmented response to alkylating chemotherapy. The ability of MGMT to counteract the effects of alkylating agents underlines its importance in therapeutic resistance.

Beyond predicting treatment response, *MGMT* promoter methylation serves as a prognostic marker. Studies indicate that patients with methylated MGMT have longer overall survival and progression-free survival rates compared to their counterparts with active MGMT expression. In their analysis, Hegi et al. [16] reported that MGMT methylation status significantly correlated with improved overall survival in patients receiving TMZ and radiation therapy.

More recent investigations have reinforced these findings. For instance, a meta-analysis by Brandner et al. [73] confirmed that methylated MGMT is linked to better clinical outcomes across various studies, asserting that *MGMT* promoter methylation influences both the efficacy of chemotherapy and the overall survival of GBM patients.

The implications of these findings for precision medicine cannot be overstated. By utilizing MGMT methylation status, clinicians can stratify patients for more personalized treatment approaches. For instance, patients with unmethylated MGMT may require alternative therapies or more aggressive treatment strategies, while those with methylated MGMT can potentially benefit from alkylating chemotherapeutics such as TMZ.

The methylation status of the *MGMT* promoter is not only predictive of response to treatment but also serves as a valuable prognostic marker in GBM and other malignancies. Its dual role underscores the importance of integrating molecular features into treatment algorithms, aligning with the principles of precision medicine.

## Emerging therapeutic strategies targeting MGMT

MGMT is a key DNA repair enzyme whose regulation has become a focal point for developing innovative therapies to tackle treatment resistance in cancers such as GBM. Advances in molecular biology have paved the way for strategies that either restore MGMT function in deficient tumors or inhibit its expression where it drives chemotherapy resistance. This section delves into these groundbreaking approaches and their potential to transform cancer therapy (Table 1).

### Restoring MGMT function in tumors

Gene therapy represents a promising strategy to reintroduce MGMT activity in tumors where it has been silenced. It was demonstrated that delivering MGMT via lentiviral vectors into glioblastoma cells restored repair capacity, increasing resistance to alkylating agents like TMZ [16]. Although reactivation could aid in preventing further genomic instability, it might also affect chemotherapy sensitivity, highlighting the importance of precise therapeutic design.

*MGMT* silencing often occurs through promoter methylation, a key epigenetic event. DNA methyltransferase (DNMT) inhibitors such as 5-azacytidine and decitabine have shown potential in reversing this silencing and restoring gene expression [53, 54]. These agents demethylate CpG islands, allowing transcriptional machinery to access the *MGMT* gene, though re-expression could paradoxically increase drug resistance, necessitating tailored use (Table 1).

Histone deacetylase inhibitors (HDACi), like vorinostat, also enhance chromatin accessibility by maintaining histone acetylation [55, 57]. Combining DNMT and HDACi has demonstrated synergistic effects, promoting MGMT reactivation and impacting tumor suppressor gene expression [5]. These epigenetic strategies hold promise but require careful clinical evaluation to balance therapy efficacy and resistance risk.

### Targeted downregulation of MGMT

RNA interference and small molecule inhibitors: In tumors where MGMT contributes to chemotherapy resistance, downregulating its expression is a potential therapeutic route. Small molecules and RNA-based tools, including siRNAs and antisense oligonucleotides, have been used to reduce MGMT levels in cancer cells, enhancing TMZ sensitivity [55]. By specifically targeting *MGMT* mRNA, these strategies can achieve tumor-specific effects while minimizing harm to normal tissues.

Combining therapies to overcome resistance: Using MGMT inhibitors alongside chemotherapy can enhance treatment outcomes. Bai et al. [2] showed that pairing O^6^-BG with TMZ improved efficacy in GBM models by inhibiting MGMT’s repair function [56]. Such combination approaches exploit DNA damage accumulation and repair inhibition, making tumors more susceptible to chemotherapy.

Integrating immunotherapy with MGMT modulation: Emerging evidence suggests that MGMT status influences tumor immune profiles. Knocking down *MGMT* might boost tumor mutational burden and enhance immune system recognition, potentially improving immune checkpoint inhibitor responses [57]. Integrating MGMT modulation with immunotherapy could pave the way for more personalized and effective cancer treatments.

### Epigenetic approaches to MGMT reactivation

Epigenetic therapy is a promising avenue in GBM, where *MGMT* promoter methylation often mediates chemotherapy resistance. By reversing silencing, these therapies could restore DNA repair capacity and modify treatment response.

DNMT inhibitors: Agents such as decitabine and azacitidine inhibit DNMTs, preventing DNA methylation and reactivating silenced genes such as *MGMT* [16, 53]. Decitabine has been shown to restore MGMT expression in previously methylated tumor cells [54]. However, this reactivation may decrease TMZ sensitivity, necessitating personalized treatment planning (Table 1).

HDACi: HDACi like vorinostat maintain acetylation of histone tails, leading to an open chromatin structure that allows transcription of silenced genes. Combining DNMT and HDACi can reactivate MGMT synergistically and enhance chemotherapy sensitivity [5]. These findings support the exploration of combination therapies targeting multiple epigenetic pathways (Table 1).

### CRISPR/Cas9 strategy for MGMT modulation

Recent advancements in epigenome-editing tools such as CRISPR-dCas9 systems offer a precise approach to modulate *MGMT* expression by targeting its promoter methylation or enhancer elements. This opens new avenues for reactivating silenced *MGMT* in normal tissue or silencing overexpressed *MGMT* in chemoresistant tumors [74]. Such programmable systems, especially when coupled with epigenetic effectors like DNMT3A or TET1 fusions, have demonstrated efficacy in preclinical models of glioblastoma and colorectal cancer [75].

Demethylation of the *MGMT* promoter: CRISPR/Cas9 can target the methylated *MGMT* promoter to reactivate its expression, increasing DNA repair and potentially sensitizing tumors to chemotherapy [2, 3]. This approach could benefit tumors with epigenetically silenced *MGMT* [16, 53].

Repairing MGMT mutations: CRISPR also allows precise correction of *MGMT* gene mutations, potentially restoring normal function and reducing chemotherapy resistance [54, 55]. Correcting dysfunctional sequences can support overall DNA repair pathways and limit mutation accumulation.

Understanding regulatory networks: CRISPR can dissect MGMT regulatory pathways by editing transcription factors like SP1 and NF-κB [57]. This capability enhances understanding of MGMT modulation and could reveal combinatorial targets for therapy [16, 56].

### Personalizing cancer therapy with multi-omics

The integration of multi-omics approaches combining methylome, transcriptome, proteome, and chromatin accessibility data is transforming our understanding of MGMT regulation and its context-dependent impact on therapy resistance. Single-cell and spatial omics further unravel the intratumoral heterogeneity of MGMT expression and its epigenetic determinants across cancer types [76] (Table 1).

Genomic profiling: Assessing *MGMT* mutations and other key oncogenic alterations like IDH1 can refine therapy choices [16]. Broader sequencing informs the overall mutational landscape, enabling tailored interventions.

Epigenomic assessment: *MGMT* promoter methylation profiling informs therapy decisions, as methylated tumors are more responsive to alkylating agents like TMZ [53]. Incorporating methylation data with other molecular profiles enhances therapy selection.

Transcriptomic insights: Examining *MGMT* and DNA repair gene expression helps predict therapy response and guides combination treatments [54]. Transcriptomics aids in identifying patients who may benefit from alkylating agents or need alternative strategies.

Customized treatment plans: Integrating multi-omics data allows clinicians to design precise therapies. Patients with methylated MGMT may benefit from alkylating agents, while those with high MGMT activity might need MGMT inhibitors or other approaches [55]. This ensures that therapy matches the tumor’s unique profile.

Balancing efficacy and toxicity: Multi-omics integration optimizes drug selection and reduces side effects. Evidence shows that patients with methylated MGMT fare better on alkylating agents, while others may require different strategies [56]. This personalized strategy aligns therapy with tumor biology, improving outcomes.

Thanks to advances in multi-omics technologies, scientists are now able to paint a much clearer picture of MGMT’s role in cancer well beyond what single methylation tests can offer. For instance, a 2016 study by Ceccarelli et al. [77] utilized integrative multi-omics analysis to identify *MGMT* promoter methylation and IDH mutation subtypes in glioblastoma, which correlated with therapy response and survival outcomes. The GLASS consortium showed that shifts in MGMT methylation and expression directly tie into drug resistance and tumor evolution in glioblastoma, revealing layers of complexity that routine biopsies miss [78]. Building on that, clinical trials like NCT04555577 and projects like PCAWG are using multi-omics data combining epigenetic, genomic, and immune profiles to tailor more precise, combination-based cancer therapies [79]. It’s a new era of biomarker-driven personalization, powered by integrated biology. Similarly, The Cancer Genome Atlas (TCGA) project incorporated genomic, transcriptomic, and methylation profiles to guide patient stratification and therapeutic development based on MGMT status. These cases highlight the translational potential of multi-omics approaches in personalizing MGMT-related cancer therapy [77, 80]. MGMT status varies across different cancers, influencing therapeutic outcomes and survival (Table 2).

**Table 2:** Comparative landscape of MGMT methylation and expression across cancer types-A pan-cancer insight into prognostic and therapeutic implications

**Cancer type**	**MGMT methylation/expression**	**Clinical relevance**
Glioblastoma [81, 82]	Hypermethylated (> 50%)	Predicts temozolomide (TMZ) response, better OS
Colorectal cancer [83]	Hypermethylated (> 50%)	Associated with MSI, chemosensitivity
Cervical cancer [6]	Hypermethylated (> 60%)	Increased risk, poor survival, and non-responsiveness to cisplatin-based chemoradiotherapy
Lung cancer [84]	Variable	Linked to prognosis, but less consistent
Head and neck cancer [85]	Hypermethylated (> 47%)	Loss of function, tumor progression, and resistance to alkylating agents
Breast cancer [14]	Hypermethylated (> 40%)	Better response to cyclophosphamide-doxorubicin followed by taxane
Pancreatic cancer [86]	Down-regulated	Increased response to streptozocin (alkylating agents)
Pituitary cancer [87]	Hypermethylated (> 40%) and down-regulated	Tumor aggressiveness and response to TMZ
Spinal glioma [88]	Hypermethylated and downregulated Unmethylated and upregulated	Better response to TMZ Response to TMZ + cisplatin
Osteosarcoma [89]	Hypermethylated and downregulated	Increased progression but better response to chemotherapy
Esophageal cancer [90]	Loss of MGMT	Promoting malignant transformation and metastatic potential
Melanoma [91]	Hypermethylated	Better response to TMZ in metastatic melanoma
Ovarian cancer [92]	Hypermethylated with down-regulation	Tumor progression and aggressiveness
Thyroid cancer [93]	Loss of MGMT	Tumor development and progression

MGMT: O^6^-methylguanine-DNA methyltransferase; MSI: microsatellite instability

Above mentioned innovative approaches highlight the dynamic interplay between MGMT expression, chemotherapy sensitivity, and treatment resistance. Integrating gene editing, epigenetic therapies, and multi-omics data can refine precision oncology and improve care for patients with MGMT-driven treatment challenges.

## Clinical relevance of MGMT across cancer types

The significance of MGMT status both at the expression and promoter methylation levels varies across cancer types, offering insights into its potential as a biomarker for diagnosis, prognosis, and therapeutic response. In glioblastoma, *MGMT* promoter hypermethylation is a well-established predictive biomarker for TMZ sensitivity and improved overall survival, making it a standard-of-care marker in neuro-oncology. Similar trends have been observed in colorectal, breast, melanoma, and pituitary cancers, where MGMT hypermethylation is associated with increased treatment responsiveness, particularly to alkylating agents.

Conversely, cancers such as cervical, esophageal, and thyroid often exhibit *MGMT* silencing or loss of function, which correlates with tumor progression, CRT resistance, and poorer clinical outcomes. In pancreatic and spinal gliomas, a more nuanced pattern emerges low MGMT levels are linked to better responses to alkylating regimens like streptozocin or combined TMZ-cisplatin therapy. Importantly, tumor-specific variability in methylation thresholds and expression patterns underscores the need for cancer-type-tailored strategies when leveraging MGMT as a biomarker (Table 2).

This comparative insight reinforces MGMT’s broad relevance while highlighting its context-dependent functional role in cancer biology and therapy optimization. Although MGMT is a key biomarker and therapeutic target in several cancers, converting lab insights into clinical success remains complex. The main hurdles include toxicity from MGMT inhibitors affecting healthy cells, difficulties delivering these drugs across the blood-brain barrier (especially for brain tumors), and tumor resistance driven by backup DNA repair mechanisms. On top of that, tests for MGMT methylation vary in accuracy, and there’s no standardized cutoff to define *MGMT* silencing across cancer types making treatment decisions tricky. Tackling these obstacles will require smarter drug designs, better diagnostic tools, and personalized treatment plans.

## Future directions

Significant progress has been made in understanding the function of MGMT, yet several avenues remain to be explored that could shape the next generation of cancer therapies. One promising area for future research is investigating MGMT’s role in immune modulation. Recent studies suggest that DNA repair pathways may influence tumor immunogenicity and immune checkpoint responses. Exploring how MGMT expression alters tumor-immune interactions could reveal opportunities to combine DNA repair inhibitors with immunotherapies to enhance anti-tumor efficacy [2, 5].

Recent studies suggest that MGMT-deficient tumors exhibit increased mutational burden, which may enhance neoantigen presentation and immune recognition. This opens the door to combining MGMT inhibition with immune checkpoint blockade to augment anti-tumor responses [2, 5]. Further investigations into MGMT’s cross-talk with TIME could redefine its utility beyond DNA repair.

Developing more advanced MGMT-targeted therapies is also essential. Although inhibitors like O^6^-BG have demonstrated potential, they often result in systemic toxicity. Future strategies may include tumor-specific delivery systems, prodrugs, or nanotechnology-based carriers to selectively suppress MGMT in cancer cells while minimizing adverse effects on healthy tissues [3].

An additional emerging direction involves the use of liquid biopsies for MGMT screening. Detecting *MGMT* promoter methylation or expression levels through blood-based biomarkers could offer a non-invasive method for real-time treatment monitoring and early detection of therapy resistance. This approach would complement existing imaging and tissue biopsy methods, particularly in tumors that are challenging to access, such as glioblastoma [15, 16]. Although promising, the application of liquid biopsy for MGMT methylation monitoring faces challenges, including the low abundance of ctDNA, variability in sample quality, and the need for high-sensitivity detection techniques like digital PCR or next-generation sequencing. Standardizing these platforms remains critical for clinical translation. Collectively, these future-focused strategies aim to personalize therapy, reduce treatment failures, and improve overall survival rates for patients with MGMT-influenced cancers.

Looking ahead, emerging technologies such as AI-driven biomarker discovery and single-cell epigenomics offer transformative potential. AI and machine learning models are increasingly used to identify predictive MGMT methylation patterns and integrate complex genomic data for treatment decision-making. In parallel, single-cell epigenomic profiling enables the study of MGMT regulation at unprecedented resolution, revealing tumor heterogeneity and uncovering resistant subpopulations within tumors. These advancements may lead to more precise, patient-specific therapies and earlier detection of resistance mechanisms [94, 95].

## Conclusions

MGMT is a pivotal DNA repair enzyme with profound implications for cancer biology, influencing tumor development, treatment response, and resistance to alkylating agents. This review has summarized the gene’s structure and regulatory mechanisms, including the impact of genetic and epigenetic modifications on its expression and therapeutic relevance. We have highlighted the role of promoter methylation, histone modifications, and ncRNAs in modulating MGMT levels, thereby affecting the efficacy of chemotherapy.

Furthermore, we explored emerging therapeutic strategies, such as gene editing, epigenetic therapies, and personalized medicine, which hold promise for overcoming MGMT-driven resistance. As research continues to advance, translating MGMT biology into clinical practice will be critical for developing targeted therapies that improve patient outcomes across a range of tumor types. Ultimately, understanding and modulating MGMT represents a cornerstone of future efforts to deliver more precise, effective, and individualized cancer care.

## Abbreviations

3′UTR: 3′ untranslated region
AP-1: activator protein 1
ATR: ataxia telangiectasia and Rad3-related protein
BCNU: carmustine
BER: base excision repair
CCNU: lomustine
CRT: chemoradiotherapy
DNMT: DNA methyltransferase
GBM: glioblastoma multiforme
H3K9me3: trimethylation of histone H3 at lysine 9
HDACi: histone deacetylase inhibitors
MDR: multidrug resistance
MGMT: O^6^-methylguanine-DNA methyltransferase
miRNAs: microRNAs
MMR: mismatch repair
MSI: microsatellite instability
ncRNAs: non-coding RNAs
O^6^-BG: O^6^-benzylguanine
O^6^-MeG: O^6^-methylguanine
PARP: poly(ADP-ribose) polymerase
SNPs: single-nucleotide polymorphisms
TIME: tumor immune microenvironment
TMZ: temozolomide

## Declarations

### Copyright

© The Author(s) 2025.
